# Bioactive Diphenyl Ethers and Isocoumarin Derivatives from a Gorgonian-Derived Fungus *Phoma* sp. (TA07-1)

**DOI:** 10.3390/md15060146

**Published:** 2017-05-25

**Authors:** Ting Shi, Jun Qi, Chang-Lun Shao, Dong-Lin Zhao, Xue-Mei Hou, Chang-Yun Wang

**Affiliations:** 1 Key Laboratory of Marine Drugs, School of Medicine and Pharmacy, Ocean University of China, the Ministry of Education of China, Qingdao 266003, China; shiting_jia@126.com (T.S.); qljbgreat@163.com (J.Q.); shaochanglun@163.com (C.-L.S.); zhaodonglin@caas.cn (D.-L.Z.); houxuemei_1990@163.com (X.-M.H.); 2 Laboratory for Marine Drugs and Bioproducts, Qingdao National Laboratory for Marine Science and Technology, Qingdao 266071, China; 3 Institute of Evolution & Marine Biodiversity, Ocean University of China, Qingdao 266003, China

**Keywords:** gorgonian-derived fungus, *Phoma* sp., diphenyl ether, isocoumarin, antibacterial activity

## Abstract

Three new diphenyl ether derivatives—phomaethers A–C (**1**–**3**) and five known compounds—including a diphenyl ether analog, 2,3′-dihydroxy-4-methoxy-5′,6-dimethyl diphenyl ether (**4**); and four isocoumarin derivatives, diaportinol (**5**), desmethyldiaportinol (**6**), citreoisocoumarinol (**7**), and citreoisocoumarin (**8**)—were isolated from a gorgonian-derived fungus *Phoma* sp. (TA07-1). Their structures were elucidated by extensive spectroscopic investigation. The absolute configurations of **1** and **2** were determined by acid hydrolysis reactions. It was the first report to discover the diphenyl glycoside derivatives from coral-derived fungi. Compounds **1**, **3**, and **4** showed selective strong antibacterial activity against five pathogenic bacteria with the minimum inhibiting concentration (MIC) values and minimum bactericidal concentration (MBC) values between 0.156 and 10.0 μM.

## 1. Introduction

**Figure 1:**
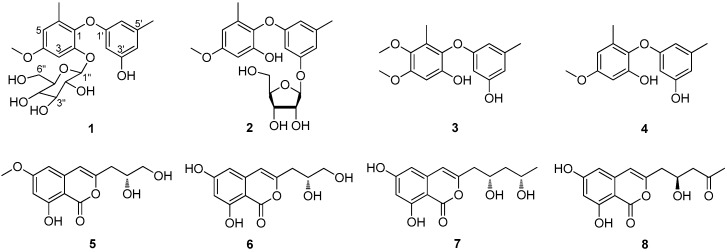
Structures of compounds **1**–**8**.

Marine microorganisms, especially marine fungi, have got more and more attention in recent years, as their outstanding abilities to produce bioactive compounds [1,2,3]. Among marine fungi, coral-derived fungi have played an important role in discovering pharmaceutical useful compounds [4]. Marine-derived *Phoma* sp. has been found to produce many novel and bioactive secondary metabolites such as cytotoxic epoxyphomalin A [5] and phomazine B [6], antifouling (+)-flavipucine [7], and antibacterial phomalevones A–C [8]. During our ongoing investigation for new bioactive compounds from gorgonian-derived fungi in the South China Sea [9,10,11,12,13], the fungal strain *Phoma* sp. (TA07-1) isolated from gorgonian *Dichotella gemmacea* attracted our attention, because its EtOAc extract of the fermentation showed significant antibacterial activity towards two Gram-positive bacteria, *Staphylococcus albus* and *S. aureus*, and three Gram-negative bacteria *Escherichia coli*, *Vibrio parahaemolyticus*, and *V. anguillarum.* Bioassay-guided separation led to the isolation of four diphenyl ether derivatives, including three new compounds, phomaethers A–C (**1**–**3**), and 2,3′-dihydroxy-4-methoxy-5′,6-dimethyl diphenyl ether (**4**) [14], together with four known isocoumarin derivatives, diaportinol (**5**) [15], desmethyldiaportinol (**6**) [16], citreoisocoumarinol (**7**) [17], and citreoisocoumarin (**8**) [17] (Figure 1). Herein we report the isolation, structure elucidation, and bioactivities of these compounds.

## 2. Results and Discussion

**Figure 2:**
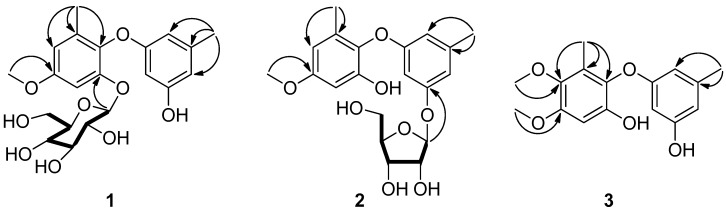
^1^H-^1^H COSY (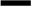) and HMBC (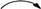) correlations for compounds **1**–**3**.

Phomaether A (**1**) was isolated as a colorless, amorphous powder. The molecular formula of C_21_H_26_O_9_ was determined by HRESIMS that displayed the [M + Na]^+^ peak at *m*/*z* 445.1474 (calcd. for C_21_H_26_O_9_Na, 445.1469) indicating nine degrees of unsaturation. The ^1^H NMR spectrum (Table 1) displayed five aromatic proton signals at δ_H_ 6.69 (1H, d, *J* = 2.9 Hz), 6.51 (1H, d, *J* = 2.9 Hz), 6.18 (1H, brs), 6.08 (1H, brs) and 5.93 (1H, brs), two methyls at δ_H_ 2.13 (3H, s) and 2.02 (3H, s), one methoxyl at δ_H_ 3.73 (3H, s), and a hydroxyl at δ_H_ 9.27 (1H, brs). The ^13^C NMR (Table 1) showed 12 aromatic carbon signals at δ_C_ 159.3, 158.2, 156.3, 150.9, 139.5, 135.2, 132.4, 109.4, 108.5, 106.6, 100.82, and 99.2; two methyls at δ_C_ 21.2, 16.2; and a methoxyl at δ_C_ 55.2. The NMR spectral feature indicated that **1** was a diphenyl ether derivative and very similar to 2,3′-dihydroxy-4-methoxy-5′,6-dimethyl diphenyl ether (**4**) [14]. The difference between these two compounds was the presence of a hexose residue in **1**. The signals of the hexose residue in ^1^H NMR and ^13^C NMR displayed five oxymethines (δ_H_ 4.82, δ_C_ 100.76; δ_H_ 3.30, δ_C_ 77.3; δ_H_ 3.20, δ_C_ 76.8; δ_H_ 3.06, δ_C_ 73.2; δ_H_ 3.08, δ_C_ 69.8), one oxymethylene (δ_H_ 3.68, 3.40, δ_C_ 60.8), and four hydroxyls (δ_H_ 5.04, 5.04, 4.68, 4.60), and the hexose was determined as glucopyranose comparing to the NMR data with those of flavonoid glycoside, 4′-demethylleucomin-7-*O*-β-d-glucopyranoside [18]. The correlation from H-1″ to C-2 in HMBC (Figure 2) indicated the glucosyl was linked to C-2. The relative configuration of glucopyranose in **1** was determined by the ^1^H-^1^H coupling constants. The large coupling constant between H-1″ and H-2″ (*J* = 7.8 Hz) indicated a β-configuration of the glucopyranose [19]. The configuration of the glucopyranose was determined as d-glucopyranose by comparing the rotation of its acid hydrolysate (${\left[\alpha \right]}_{D}^{25}$ +48.0 (*c* 0.04, H_2_O)) with that of the standard d-glucopyranose (${\left[\alpha \right]}_{D}^{25}$ +54.0 (*c* 0.15, H_2_O)). Accordingly, **1** was determined as 2-*O*-β-d-glucopyranose-3′-hydroxy-4-methoxy-5′,6-dimethyl diphenyl ether, and was named phomaether A.

Phomaether B (**2**) was isolated as a light brown, amorphous powder. The molecular formula was assigned as C_20_H_24_O_8_ (nine degrees of unsaturation) by its HRESIMS data. Detailed inspection of the NMR data (Table 1) of **2** with those of **1** revealed that these two compounds were very similar, except for the sugar moiety. The sugar residue in **2** was defined as a ribose by comparing the NMR data (Table 1) with those of naphthyl ribofuranoside, isotorachrysone-6-*O*-α-d-ribofuranoside [20], chromene glycoside, and sterin A [21]. The key HMBC correlation from H-1″ to C-3′ (Figure 2) established the connection between the ribose and diphenyl ether moiety. The coupling constant of anomeric proton H-1″ (*J* = 4.5 Hz) in **2** was found to close to that in methyl-α-d-ribofuranoside (*J* = 4.3 Hz) [22], indicating an α-ribose in **2**. The α-ribose was determined as d-configuration by comparing the optical rotation data of the acid hydrolysate of **2** with that of the standard d-ribose (${\left[\alpha \right]}_{D}^{25}$ −23.0 (*c* 0.03, H_2_O) vs. ${\left[\alpha \right]}_{D}^{25}$ −38.7 (*c* 0.10, H_2_O)). From above, **2** was determined as 2-hydroxy-3′-*O*-α-d-ribofuranoside-4-methoxy-5′,6-dimethyl diphenyl ether, and named phomaether B.

A literature survey revealed that the diphenyl glycoside derivatives were rare in marine natural products. To the best of our knowledge, a diphenyl glycoside was found from a sponge-derived fungus *Metarhizium anisopliae* [23]. In present study, the diphenyl glycoside derivatives were reported for the first time isolated from coral-derived fungi.

Phomaether C (**3**) was obtained as a colorless powder with a molecular formula C_16_H_18_O_5_, requiring eight degrees of unsaturation. The NMR data (Table 1) indicated that **3** is very similar to 2,3′-dihydroxy-4-methoxy-5′,6-dimethyl diphenyl ether (**4**) [14]. The only difference was the presence of an additional methoxyl in **3**. The correlation from 5-OMe to C-5 in HMBC (Figure 2) indicated that the additional methoxyl was anchored at C-5. Thus, **3** was determined as 2,3′-dihydroxy-4,5-dimethoxy-5′,6-dimethyl diphenyl ether, and named phomaether C.

The structures of **4**, **5**, **6**, **7**, and **8** were determined as 2,3′-dihydroxy-4-methoxy-5′,6-dimethyl diphenyl ether [14], diaportinol [15], desmethyldiaportinol [16], citreoisocoumarinol [17], citreoisocoumarin [17], respectively, by comparing their NMR data with those in the literature.

All the isolated compounds (**1**–**8**) were evaluated for their antibacterial activity against a panel of pathogenic bacteria, including two Gram-positive bacteria, *S. albus* and *S. aureus*, and three Gram-negative bacteria *E. coli*, *V. parahaemolyticus*, and *V. anguillarum* (Table 2). Compound **1** exhibited remarkable antibacterial activity against *S. albus*, *S. aureus*, *E. coli*, and *V. parahaemolyticus* with MIC values ranging from 0.312 to 0.625 μM and MBC values from 0.625 to 2.50 μM. Compound **3** showed strong antibacterial activity to *S. albus, S. aureus*, and *E. coli* with MIC values ranging from 0.312 to 1.25 μM and MBC values from 0.625 to 5.00 μM. It was notable that compound **4** showed strong antibacterial activity to all of the tested pathogenic bacteria, with MIC and MBC values ranging from 0.156 to 5.00 μM.

Compounds **1**–**8** were also tested for their lethality to the brine shrimp, *Artemia salina*. Compounds **1**, **3**, and **4** showed moderate lethality to the brine shrimp *A. salina* with the LC_50_ values ranging from 14.01 ± 0.36 to 37.33 ± 0.26 μg/mL.

## 3. Experimental Section

### 3.1. General Experimental Procedures

Optical rotations were measured on a JASCO P-1020 digital polarimeter (JASCO Corporation, Tokyo, Japan). UV spectra were recorded using a Milton Roy spectrophotometer (Milton Roy, New York, NY, USA). IR spectra were recorded on a Nicolet-Nexus-470 spectrophotometer using KBr pellets (Thermo Electron, Waltham, MA, USA). NMR spectra were recorded on a JEOL Eclips-600 spectrometer (JEOL, Tokyo, Japan) at 600 MHz for ^1^H and 150 MHz for ^13^C in CD_3_OD or DMSO-*d*_6_. Chemical shifts δ were recorded in ppm, using TMS as internal standard. ESIMS and HRESIMS spectra were measured on a Micromass Q-TOF spectrophotometer (Waters Corp., Manchester, UK) and a Thermo Scientific LTQ Orbitrap XL spectrometer (Thermo Fisher Scientific, Bremen, Germany), respectively. HPLC separation was performed using a Hitachi LA-2000 prep-HPLC system (Hitachi High Technologies, Tokyo, Japan) coupled with a Hitachi L-2455 photodiodearray detector (Hitachi High Technologies, Tokyo, Japan). A Kromasil C_18_ semi-preparative HPLC column (250 × 10 mm, 5 μm) (Eka Nobel, Bohus, Sweden) was used. Silica gel (200–300 mesh; Qingdao Marine Chemical Group Co., Qingdao, China) and Sephadex LH-20 (Amersham Biosciences Inc., Piscataway, NJ, USA) were used for column chromatography. Precoated silica gel GF254 plates (Yantai Zifu Chemical Group Co., Yantai, China) were used for thin layer chromatography (TLC).

### 3.2. Fungal Materials

The fungus *Phoma* sp. (TA07-1) was isolated from a piece of fresh tissue from the inner part of the gorgonian *Dichotella gemmacea* (GX-WZ-2008003-4), collected from Weizhou coral reef in the South China Sea in September 2008. The strain was deposited in the Key Laboratory of Marine Drugs, the Ministry of Education of China, School of Medicine and Pharmacy, Ocean University of China, Qingdao, China, with the GenBank (NCBI) accession number KY556682.

### 3.3. Extraction and Isolation

The fungal strain *Phoma* sp. (TA07-1) was fermented in a rice medium in 50 Erlenmeyer flasks (500 mL) at 28 °C for four weeks. Each flask contained rice (Liaoyang City Jiapin Rice Co., LTD., Liaoyang, China) 80 g, water 120 mL and sea salt (Qingdao Salt Industry Co., LTD., Qingdao, China) 2.0 g. The cultivated solid medium was extracted repeatedly with EtOAc (3 × 300 mL for each flask). The combined EtOAc layer was evaporated to dryness under reduced pressure to afford a residue (10.0 g). The residue (10.0 g) was subjected to vacuum liquid chromatography (VLC) on silica gel using step gradient elution with EtOAc–petroleum ether (PE) (0–100%) and then with MeOH–EtOAc (0–100%) to afford nine fractions (Fr. 1–Fr. 9). Fr. 4 was first isolated by column chromatography (CC) on silica gel eluted with PE–EtOAc (*v*/*v*, 8:2), then subjected to Sephadex LH-20 CC with PE–CHCl_3_–MeOH (*v*/*v*/*v*, 2:1:1), and further purified by using semi-preparative HPLC on an ODS column (Kromasil C18, 250 × 10 mm, 5 μm, 2 mL/min) eluted with 65% MeOH–H_2_O to give compound **4** (30.0 mg). Fr. 5 was separated on silica gel CC eluting with PE–EtOAc (*v*/*v*, 7:3), then isolated on Sephadex LH-20 CC with PE–CHCl_3_–MeOH (*v*/*v*/*v*, 2:1:1), and further purified on HPLC eluted with 60% MeOH–H_2_O to obtain compound **3** (5.0 mg). Fr. 8 was eluted with CHCl_3_–MeOH (*v*/*v*, 15:1) on silica gel CC, then eluted with CHCl_3_–MeOH (*v*/*v*, 1:1) on Sephadex LH-20 CC, and further purified on HPLC with 40% MeOH–H_2_O for **6** (6.0 mg), **7** (7.0 mg) and **8** (5.0 mg), 45% MeOH–H_2_O for **1** (5.0 mg) and **5** (6.0 mg), and 55% MeOH–H_2_O for **2** (3.5 mg).

Phomaether A (**1**): colorless, amorphous powder; ${\left[\alpha \right]}_{D}^{25}$ −2.1 (*c* 0.40, MeOH); UV (MeOH) λ_max_: 202, 228, 278 nm; IR (KBr) ν_max_ 3470, 2965, 1565, 1475, 1320, 1165, 980 cm^−^^1^; ^1^H and ^13^C NMR data, see Table 1; ESIMS *m*/*z* 445.1 [M + Na]^+^, 867.2 [2M + Na]^+^; HRESIMS *m*/*z* 445.1474 [M + Na]^+^ (calcd. for C_21_H_26_O_9_Na, 445.1469).

Phomaether B (**2**): light brown, amorphous powder; ${\left[\alpha \right]}_{D}^{25}$ +8.0 (*c* 0.20, MeOH); UV (MeOH) λ_max_: 204, 227, 278 nm; IR (KBr) ν_max_ 3312, 2956, 1566, 1477, 1332, 1167, 970 cm^−^^1^; ^1^H and ^13^C NMR data, see Table 1; ESIMS *m*/*z* 415.1 [M + Na]^+^, 807.1 [2M + Na]^+^; HRESIMS *m*/*z* 415.1370 [M + Na]^+^ (calcd. for C_20_H_24_O_8_Na, 415.1363).

Phomaether C (**3**): colorless powder; UV (MeOH) λ_max_: 207, 226, 281 nm; IR (KBr) ν_max_ 3422, 3310, 2944, 1578, 1465, 1298, 1165, 975 cm^−^^1^; ^1^H and ^13^C NMR data, see Table 1; ESIMS *m*/*z* 290.9 [M + H]^+^, 603.0 [2M + Na]^+^; HRESIMS *m*/*z* 291.1168 [M + H]^+^ (calcd. for C_16_H_19_O_5_, 291.1227).

### 3.4. Methanolysis of Compound ***1***

Compound **1** (2.0 mg) was dissolved in 5% HCl–MeOH (5 mL) and refluxed at boiled temperature for 2 h. The reaction mixture was neutralized and evaporated to give the residue. Then, the residue was extracted by 50% EtOAc–H_2_O to obtain aglycone. The configuration of d-glucoside was determined by comparing its rotation with that of the authentic sample.

### 3.5. Methanolysis of Compound ***2***

The aglycone of compound **2** was obtained by the same method for that of compound **1**. The configuration of d-ribose was determined by comparing its rotation with that of a standard sample.

### 3.6. Biological Assays

The antibacterial activity of compounds was evaluated by the conventional broth dilution assay [24,25]. The MICs were tested in 96-well microtiter plates, and the concentrations of the compounds were serial double dilution which were certain in each well. The MICs were determined as the lowest concentrations at which no growth was observed. The MBCs were determined by transferring approximately 0.0015 mL from each well of the microtiter plate with the MIC 2000 inoculator to a petri dish (15 by 150 mm) containing solid LB culture. The plates were incubated at 35 °C for 48 h. The MBCs were read as the lowest concentrations of compounds that prevented growth of more than one colony on subculture. The test range of compounds **1**–**8** was 0.039–20.0 μM and the test range of positive control was 0.010–10.0 μM. Five bacterial strains *S. albus* (ATCC 23361), *S. aureus* (ATCC 27154), *E. coli* (ATCC 25922), *V. parahaemolyticus* (ATCC 17802), and *V. anguillarum* (ATCC 19109) were used, and ciprofloxacin was used as a positive control. 

The lethality to the brine shrimp *A. salina* was tested according to the method in literature [26]. The brine shrimp *A. salina* eggs (Tianjin Red Sun Aquaculture Co., LTD., Tianjin, China) were incubated in nature seawater from Yellow Sea in Qingdao, China and oxygenated with an aquarium pump at 25 °C for 48 h. The nauplii of brine shrimp were separated from the eggs in small beakers containing sea water. The test compounds were dissolved in DMSO and the serial diluted concentration ranges of the compounds **1**–**8** were 0.625–100 μg/mL. In 24-well microplates, 15–20 brine shrimp in each well were incubated with the test compounds for 24 h. The lethality rates were observed and the LC_50_ values were calculated by Probit analysis.

## 4. Conclusions

In summary, three new diphenyl ether derivatives—phomaethers A–C (**1**–**3**) together with five known compounds—2,3′-dihydroxy-4-methoxy-5′,6-dimethyl diphenyl ether (**4**), diaportinol (**5**), desmethyldiaportinol (**6**), citreoisocoumarinol (**7**), and citreoisocoumarin (**8**)—were isolated from a gorgonian-derived *Phoma* sp. fungus collected from the South China Sea. It was the first example of diphenyl glycoside derivatives obtained from coral-derived fungi. Diphenyl ether derivatives **1**, **3**, and **4** showed strong antibacterial activity, suggesting that they might have potential to be developed as antibacterial agents.

## Figures and Tables

**Table 1:** NMR spectroscopic data (600/150 MHz) for compounds **1**–**3**.

Position	1, δ_C_ Type	1, δ_H_ Mult. (*J* in Hz)	2, δ_C_ Type	2, δ_H_ Mult. (*J* in Hz)	3, δ_C_ Type	3, δ_H_ Mult. (*J* in Hz)
1	135.2 C		135.6 C		134.7 C	
2	150.9 C		151.8 C		147.5 C	
3	100.82 CH	6.69, d (2.9)	101.4 CH	6.35, d (2.9)	100.0 CH	6.49, s
4	156.3 C		158.7 C		151.7 C	
5	108.5 CH	6.51, d (2.8)	107.8 CH	6.31, d (2.9)	141.3 C	
6	132.4 C		134.0 C		127.2 C	
1′	159.3 C		160.7 C		160.8 C	
2′	99.2 CH	5.93, brs	102.6 CH	6.39, brs	100.3 CH	6.02, brs
3′	158.2 C		159.8 C		159.4 C	
4′	109.4 CH	6.18, brs	111.7 CH	6.60, brs	110.4 CH	6.24, brs
5′	139.5 C		141.3 C		141.4 C	
6′	106.6 CH	6.08, brs	110.4 CH	6.29, brs	108.1 CH	6.15, brs
1″	100.76 CH	4.82, d (7.8)	102.3 CH	5.55, d (4.5)		
2″	73.2 CH	3.06, m	73.4 CH	4.13, dd (6.4, 4.5)		
3″	76.8 CH	3.20, dd (8.9, 8.8)	71.2 CH	4.06, dd (6.5, 3.2)		
4″	69.8 CH	3.08, m	87.5 CH	4.10, dt (3.5, 3.5)		
5″	77.3 CH	3.30, ddd (8.4, 6.4, 2.0)	63.2 CH_2_	3.69, dd (12.1, 3.4)		
				3.63, dd (12.2, 3.8)		
6″	60.8 CH_2_	3.68, d (11.0)				
		3.40, d (11.0)				
4-OMe	55.2 OCH_3_	3.73, s	55.8 OCH_3_	3.74, s	56.4 OCH_3_	3.81, s
5-OMe					61.0 OCH_3_	3.70, s
6-Me	16.2 CH_3_	2.02, s	16.5 CH_3_	2.03, s	9.8 CH_3_	1.98, s
3′-OH		9.27, brs				
5′-Me	21.2 CH_3_	2.13, s	21.8 CH_3_	2.24, s	21.6 CH_3_	2.18, s
2″-OH		5.04, brs				
3″-OH		5.04, brs				
4″-OH		4.68, d (5.2)				
6″-OH		4.60, brs				

Compound **1** was measured in DMSO-*d*_6_, Compounds **2** and **3** were measured in CD_3_OD.

**Table 2:** Antibacterial activity of compounds **1**, **3**, and **4**.

Compounds	MIC/MBC (μM)					Test Ranges
	*S. albus*	*S. aureus*	*E. coli*	*V. parahaemolyticus*	*V. anguillarum*	
**1**	0.312/0.625	0.625/0.625	0.625/1.25	0.625/2.50	–/–	0.039–20.0
**3**	0.625/1.25	0.312/0.625	1.25/5.00	–/–	10.0/–	0.039–20.0
**4**	0.312/0.312	0.156/0.312	0.156/0.156	0.312/0.312	2.50/5.00	0.039–20.0
Ciprofloxacin	0.312/0.312	0.156/0.156	0.156/0.156	0.156/0.156	0.156/0.156	0.010–10.0

“–” means no activity; each experiment of the activity bioassays has been repeated three times and the results were same.

## Supplementary Materials

The NMR and MS spectra of **1**, **2**, and **3** are available on line at www.mdpi.com/1660-3397/15/6/146/s1 in Figures S1–S21.
