# Supplementary material for: Bioactive Diphenyl Ethers and Isocoumarin Derivatives from a Gorgonian-Derived Fungus Phoma sp. (TA07-1)
Source: Mar Drugs. 2017 May 25;15(6):146. doi: 10.3390/md15060146 (PMC5484096; doi:10.3390/md15060146)
Supplement: Supplementary file 1 [file marinedrugs-15-00146-s001.pdf]

Supplementary Materials: Bioactive Diphenyl Ether Derivatives and Isocoumarin Derivatives from Gorgonian-Derived Fungus *Phoma* sp.

Ting Shi, Jun Qi, Chang-lun Shao, Dong-lin Zhao, Xue-mei Hou and Chang-yun Wang*

Figure S1. ^{1}H NMR spectrum of compound **1** (DMSO-d_6).
Figure S2. ^{13}C NMR spectrum of compound **1** (DMSO-d_6).
Figure S3. COSY spectrum of compound **1** (DMSO-d_6).
Figure S4. HMQC spectrum of compound **1** (DMSO-d_6).
Figure S5. HMBC spectrum of compound **1** (DMSO-d_6).
Figure S6. ESIMS spectrum of compound **1**.
Figure S7. HRESIMS spectrum of compound **1**.
Figure S8. ^{1}H NMR spectrum of compound **2** (CD_3OD).
Figure S9. ^{13}C NMR spectrum of compound **2** (CD_3OD).
Figure S10. COSY spectrum of compound **2** (CD_3OD).
Figure S11. HMQC spectrum of compound **2** (CD_3OD).
Figure S12. HMBC spectrum of compound **2** (CD_3OD).
Figure S13. ESIMS spectrum of compound **2**.
Figure S14. HRESIMS spectrum of compound **2**.
Figure S15. ^{1}H NMR spectrum of compound **3** (CD_3OD).
Figure S16. ^{13}C NMR spectrum of compound **3** (CD_3OD).
Figure S17. COSY spectrum of compound **3** (CD_3OD).
Figure S18. HMQC spectrum of compound **3** (CD_3OD).
Figure S19. HMBC spectrum of compound **3** (CD_3OD).
Figure S20. ESIMS spectrum of compound **3**.
Figure S21. HRESIMS spectrum of compound **3**.

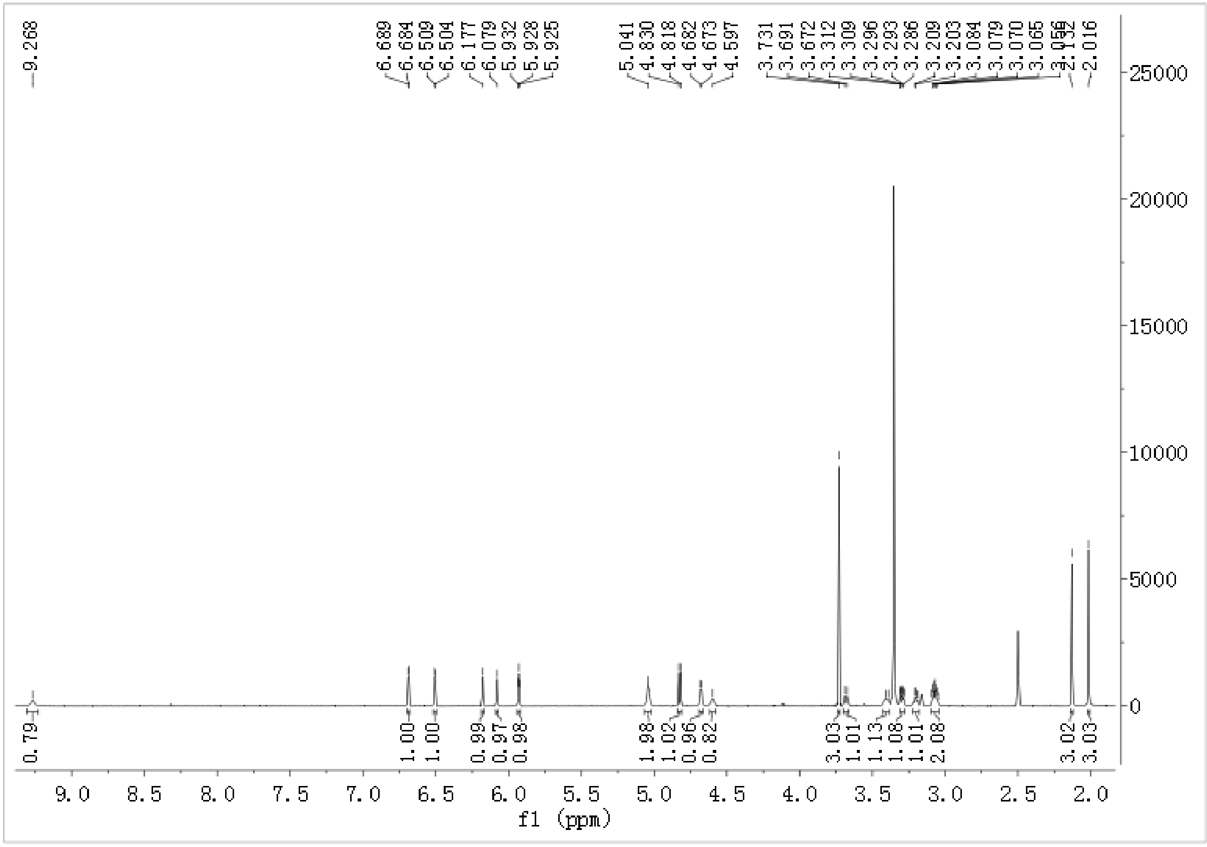

Figure S1. ^{1}H NMR spectrum of compound **1** (DMSO-d_6).

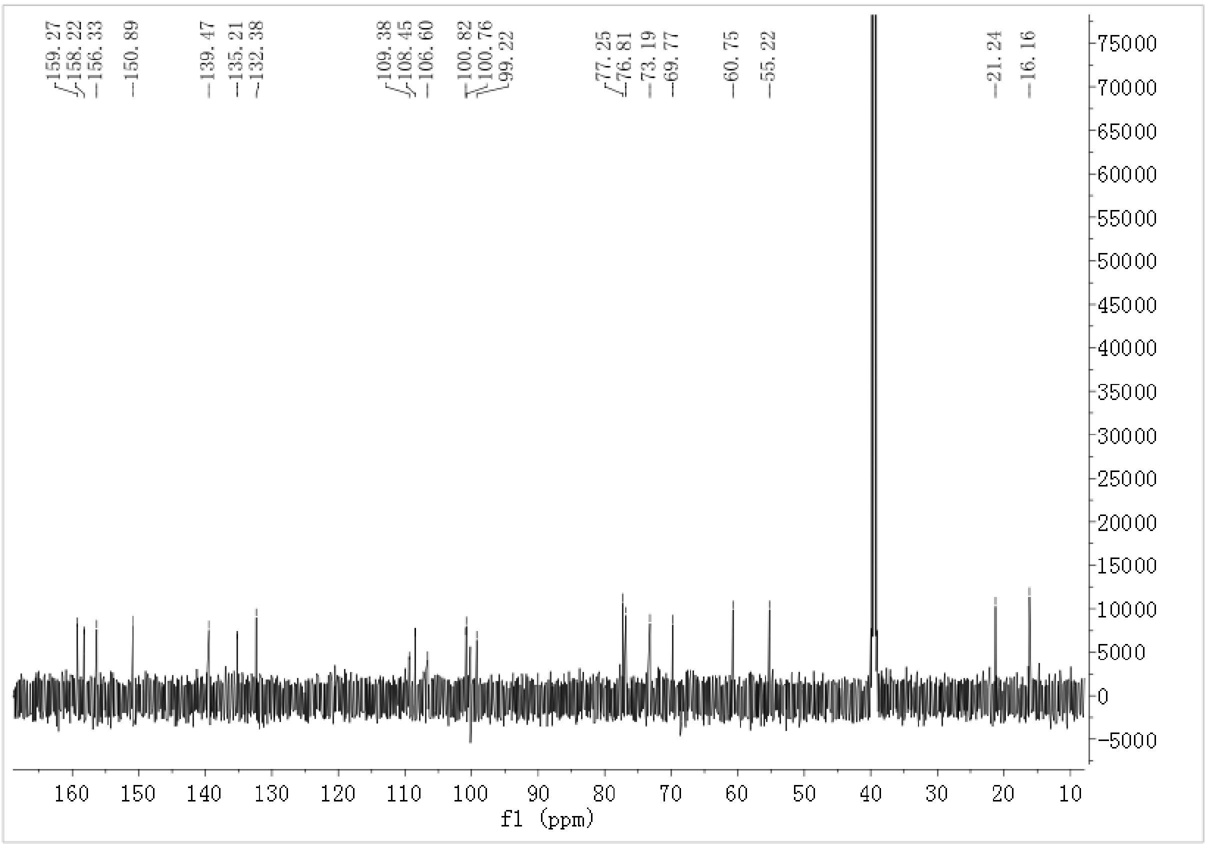

Figure S2. ^{13}C NMR spectrum of compound **1** (DMSO-d_6).

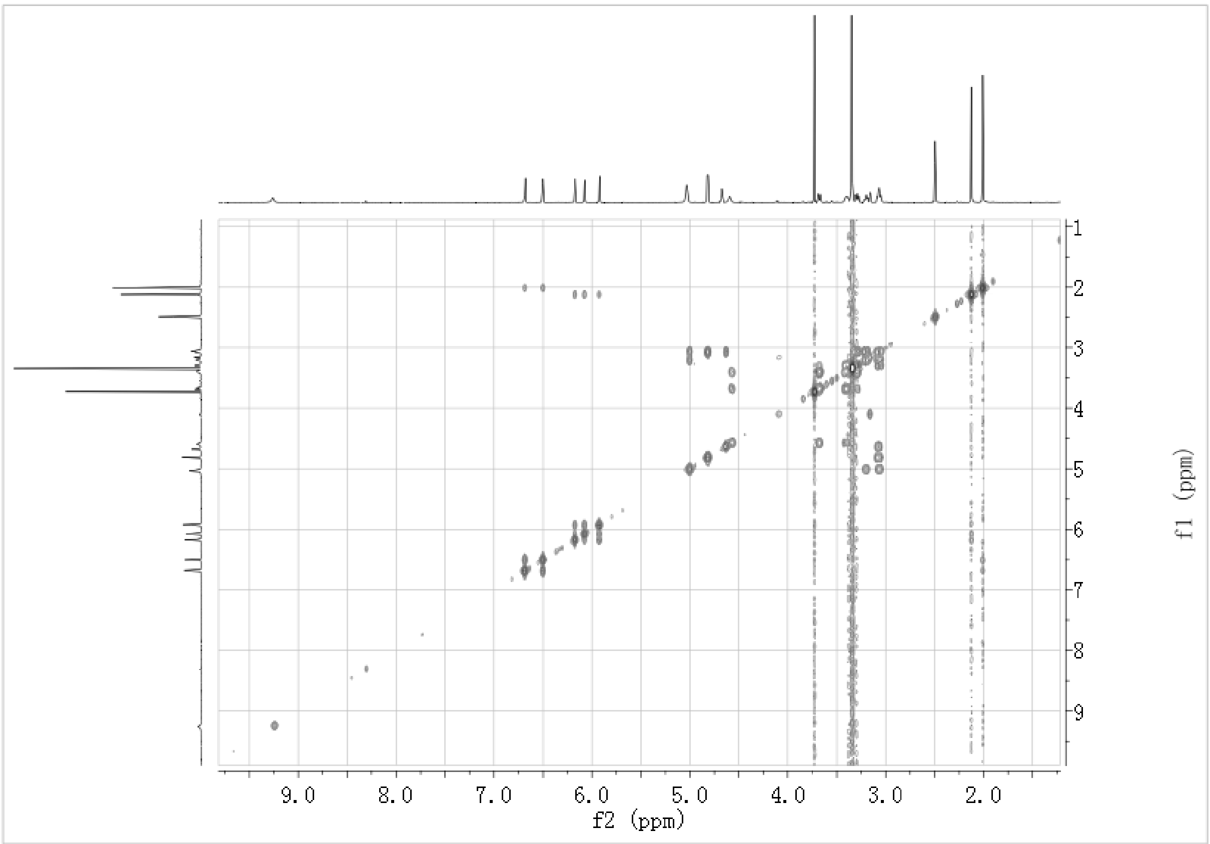

Figure S3. COSY spectrum of compound **1** (DMSO-d_6).

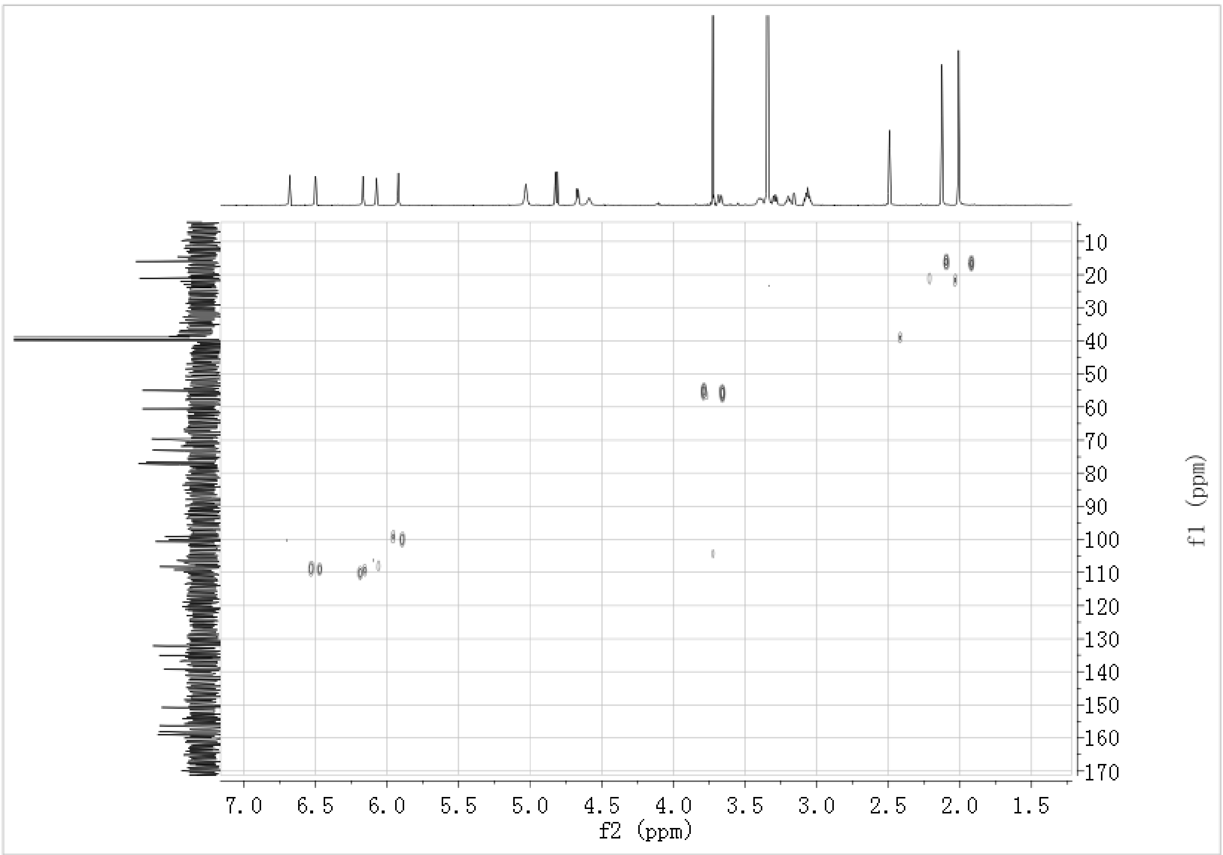

Figure S4. HMQC spectrum of compound **1** (DMSO-d_6).

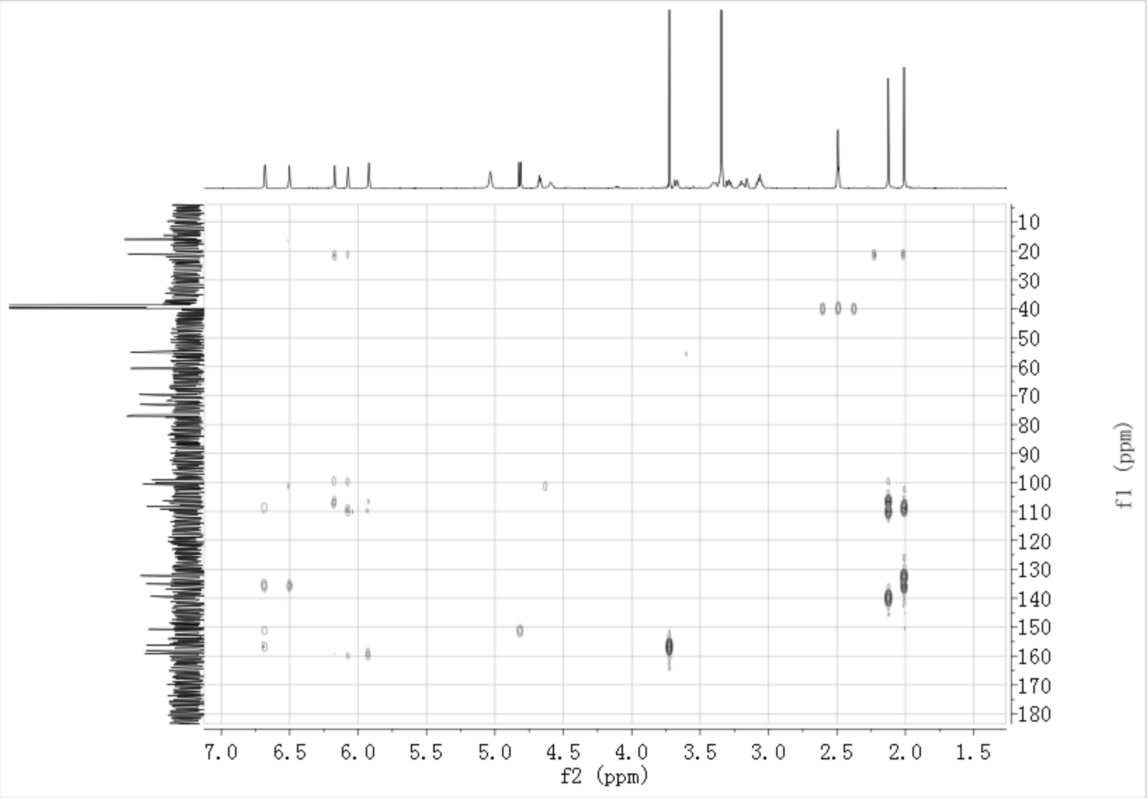

Figure S5. HMBC spectrum of compound **1** (DMSO-d_6).

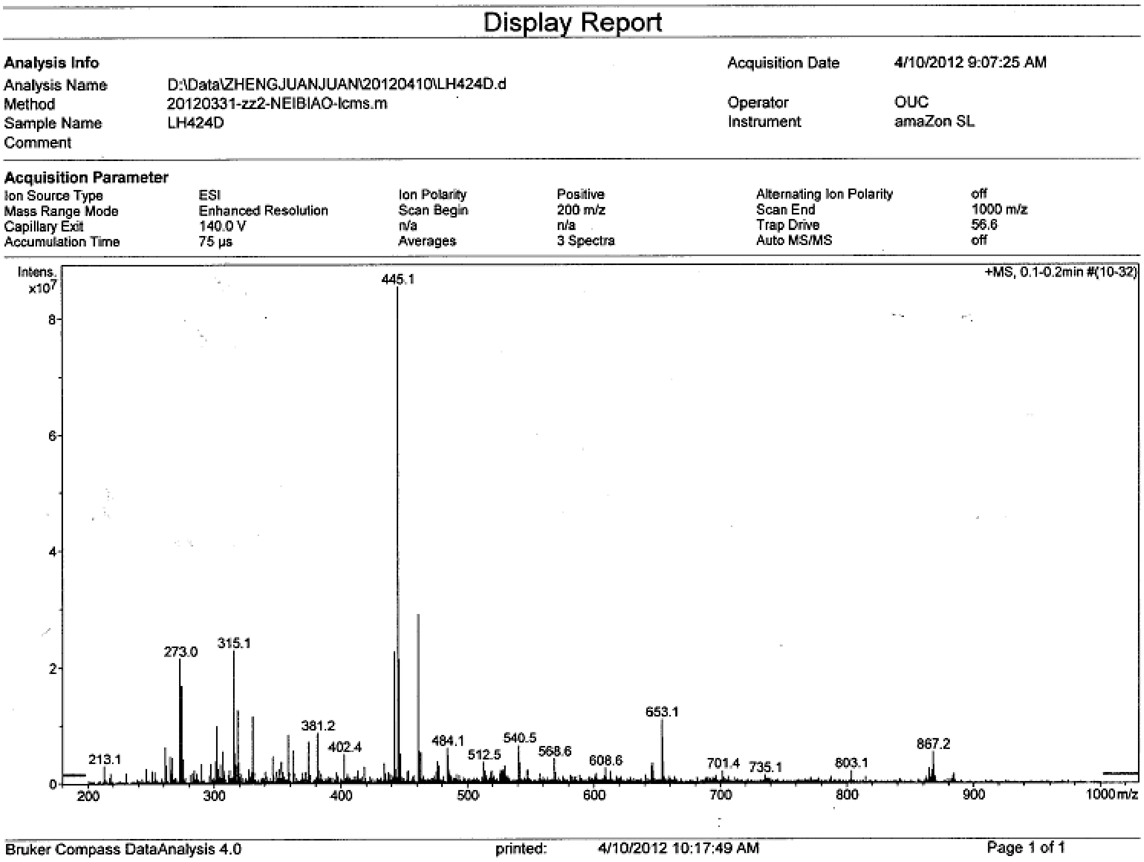

Figure S6. ESIMS spectrum of compound **1**.

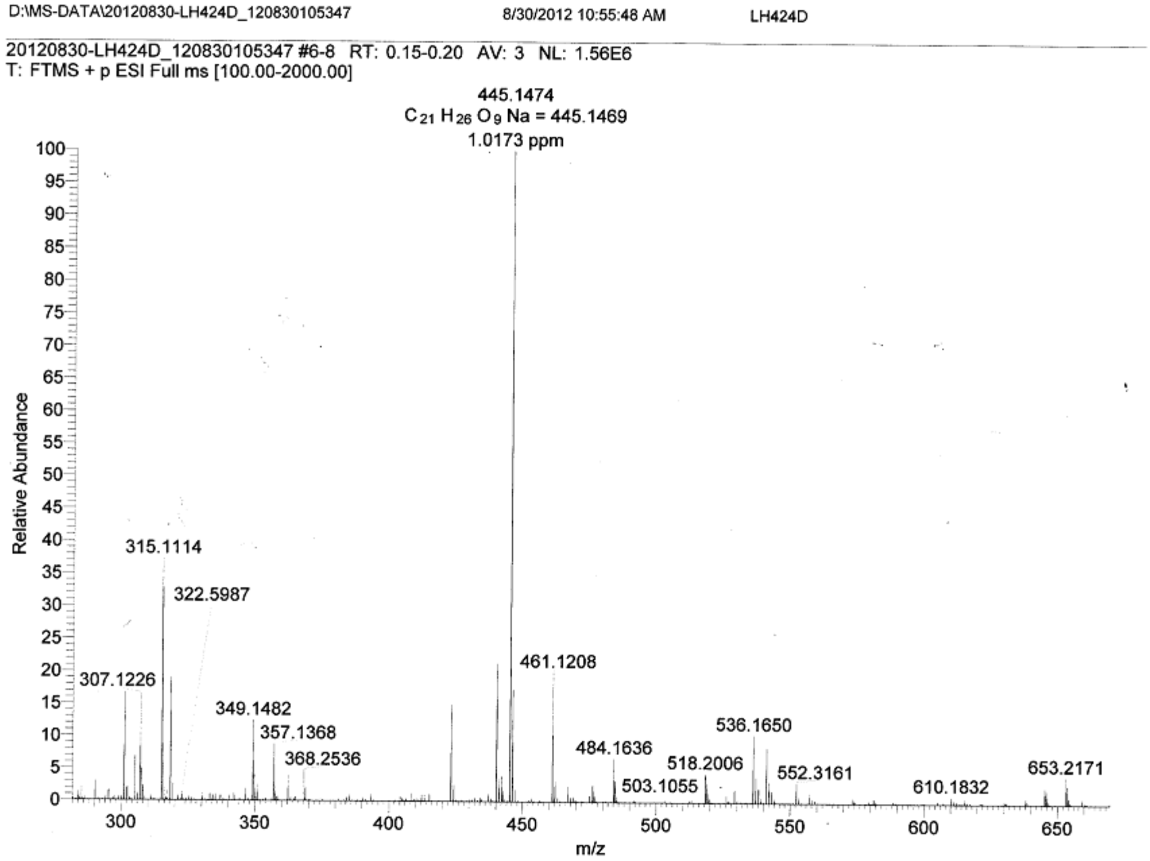

Figure S7. HRESIMS spectrum of compound **1**.

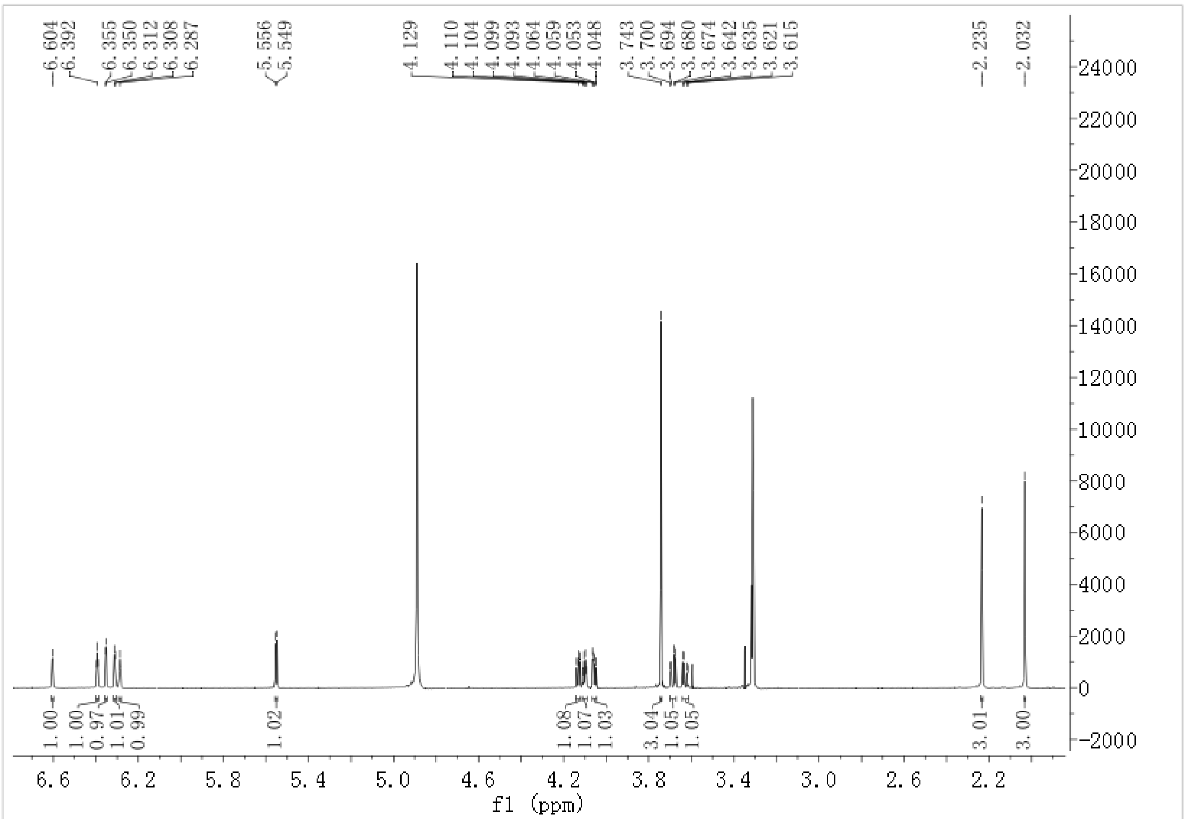

Figure S8. ^{1}H NMR spectrum of compound **2** (CD_3OD).

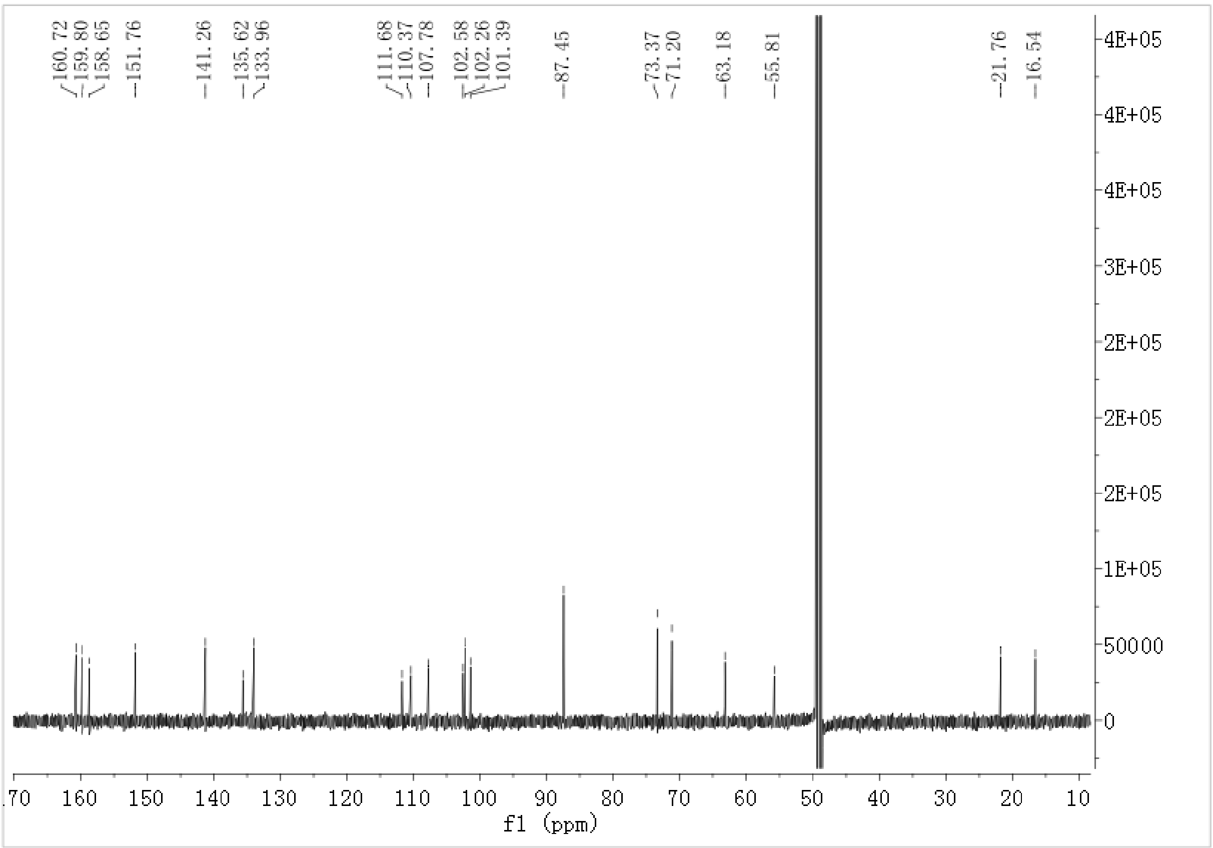

Figure S9. ^{13}C NMR spectrum of compound **2** (CD_3OD).

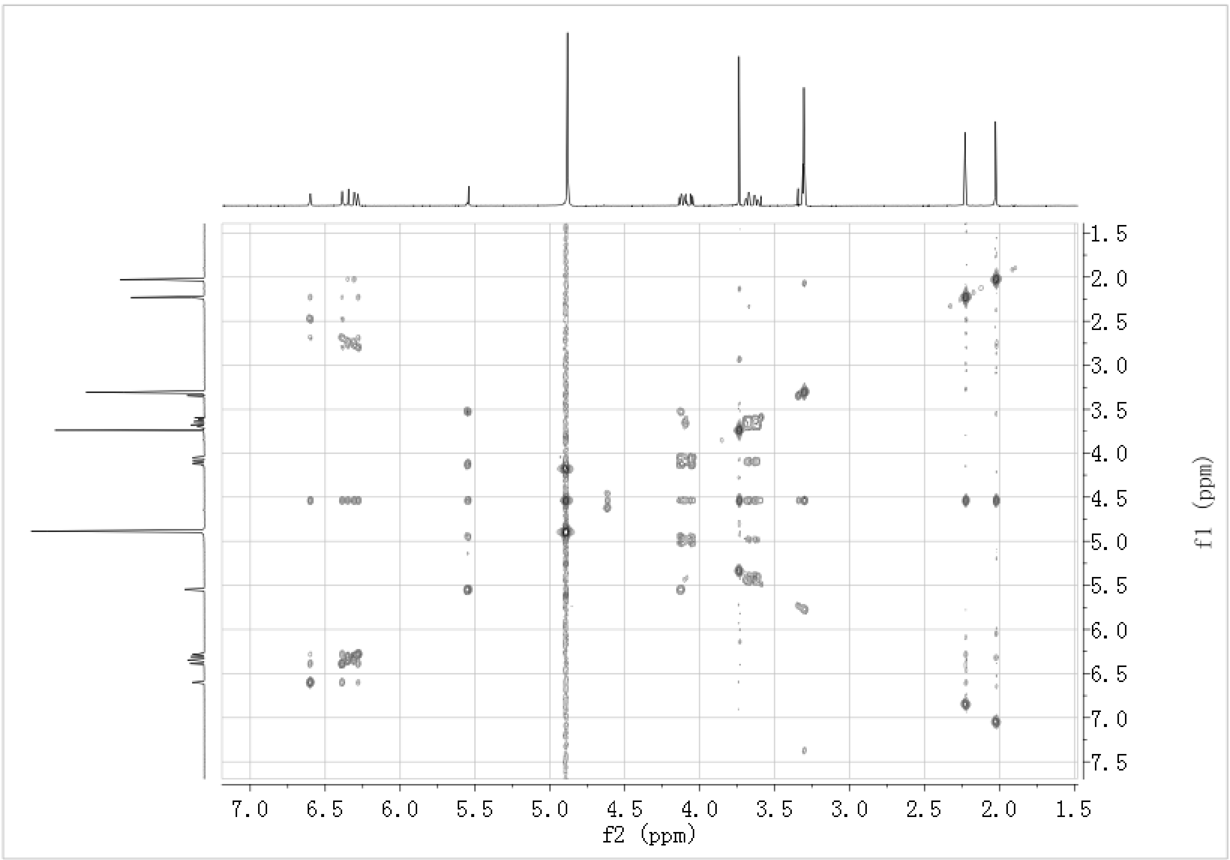

Figure S10. COSY spectrum of compound **2** (CD_3OD).

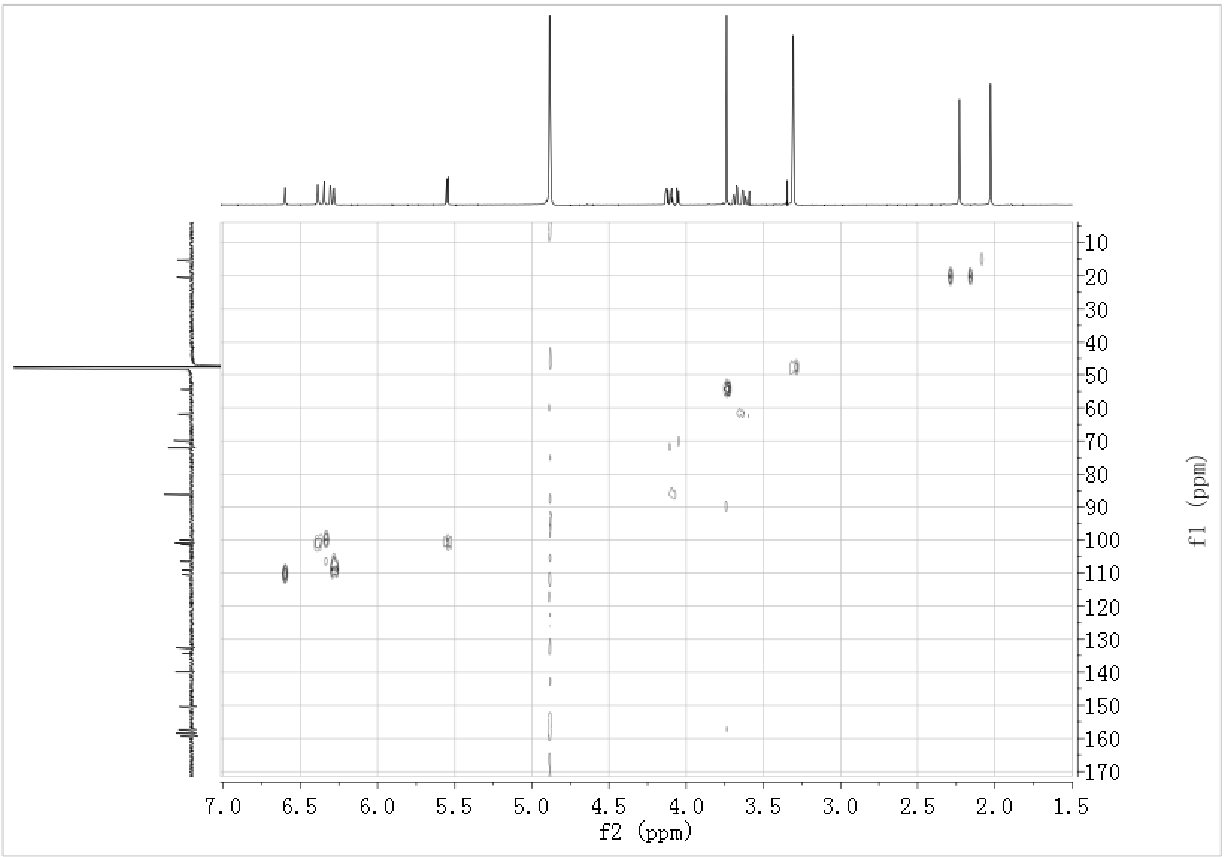

Figure S11. HMQC spectrum of compound **2** (CD_3OD).

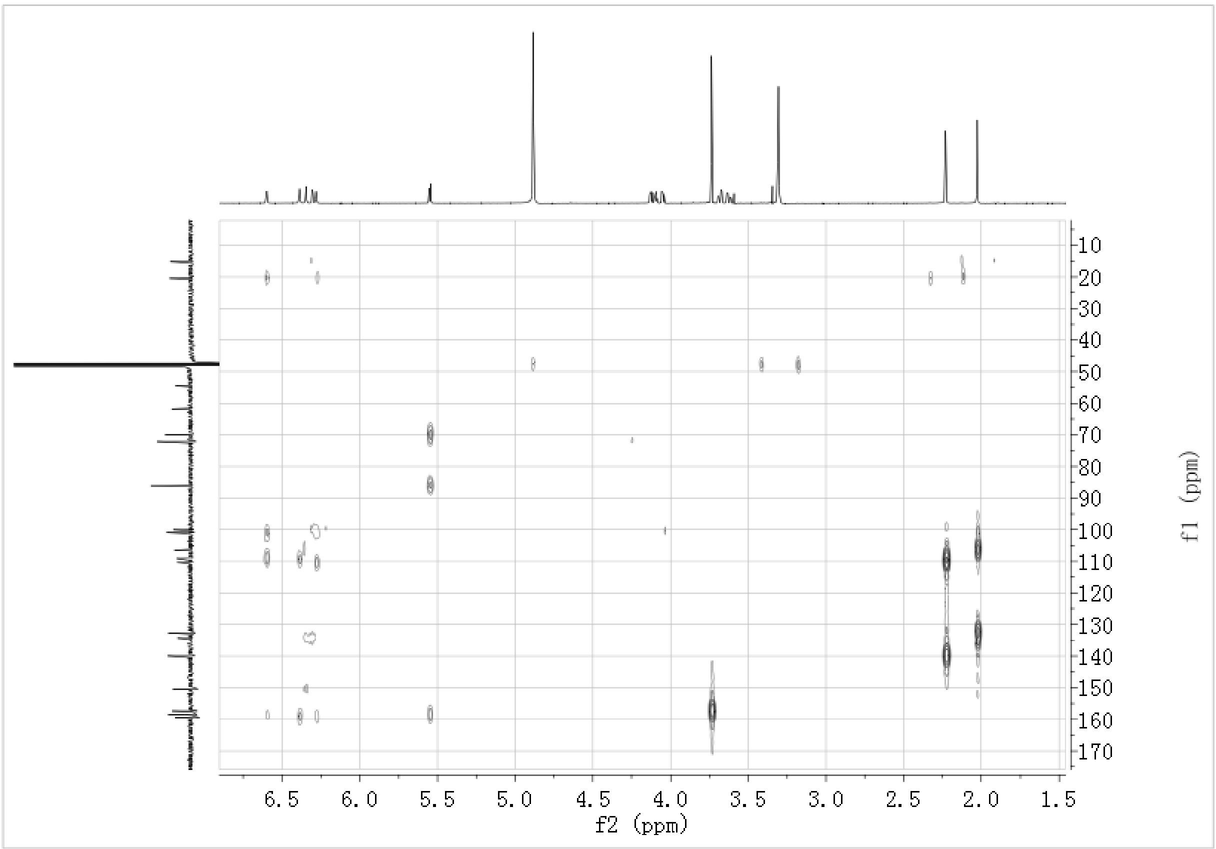

Figure S12. HMBC spectrum of compound **2** (CD_3OD).

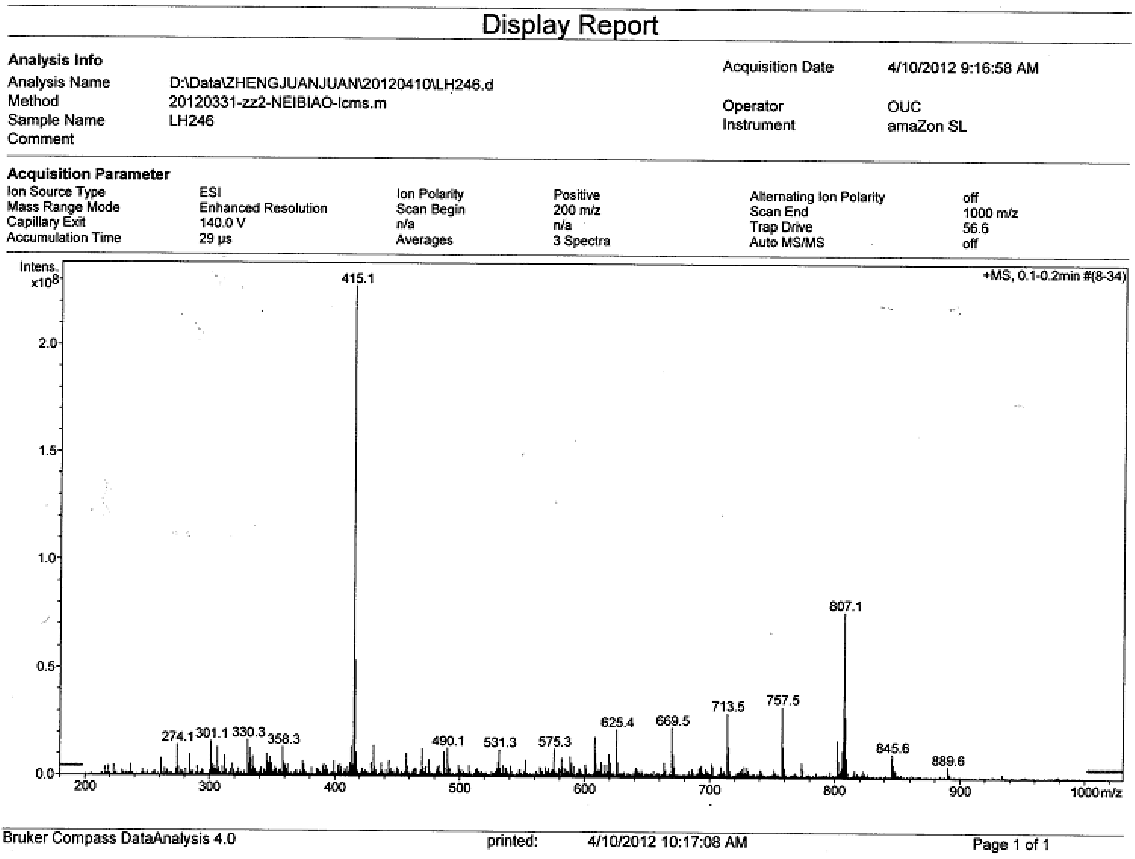

Figure S13. ESIMS spectrum of compound **2**.

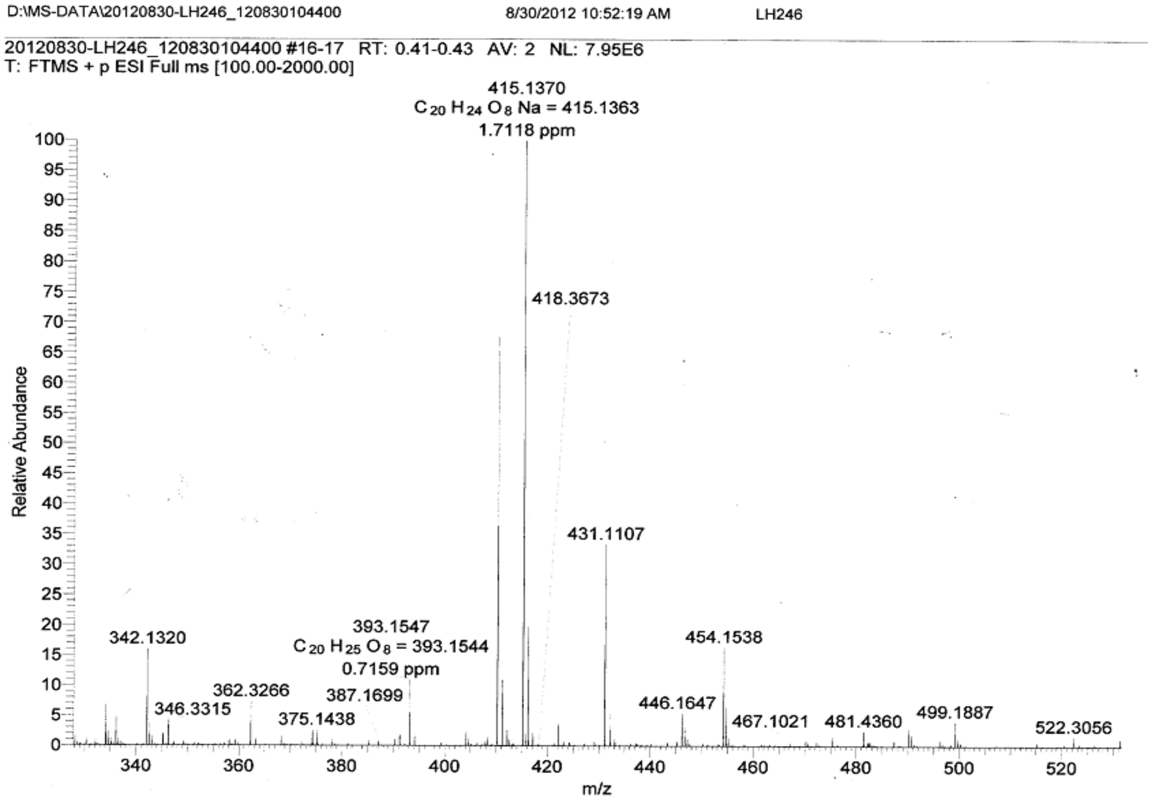

Figure S14. HRESIMS spectrum of compound **2**.

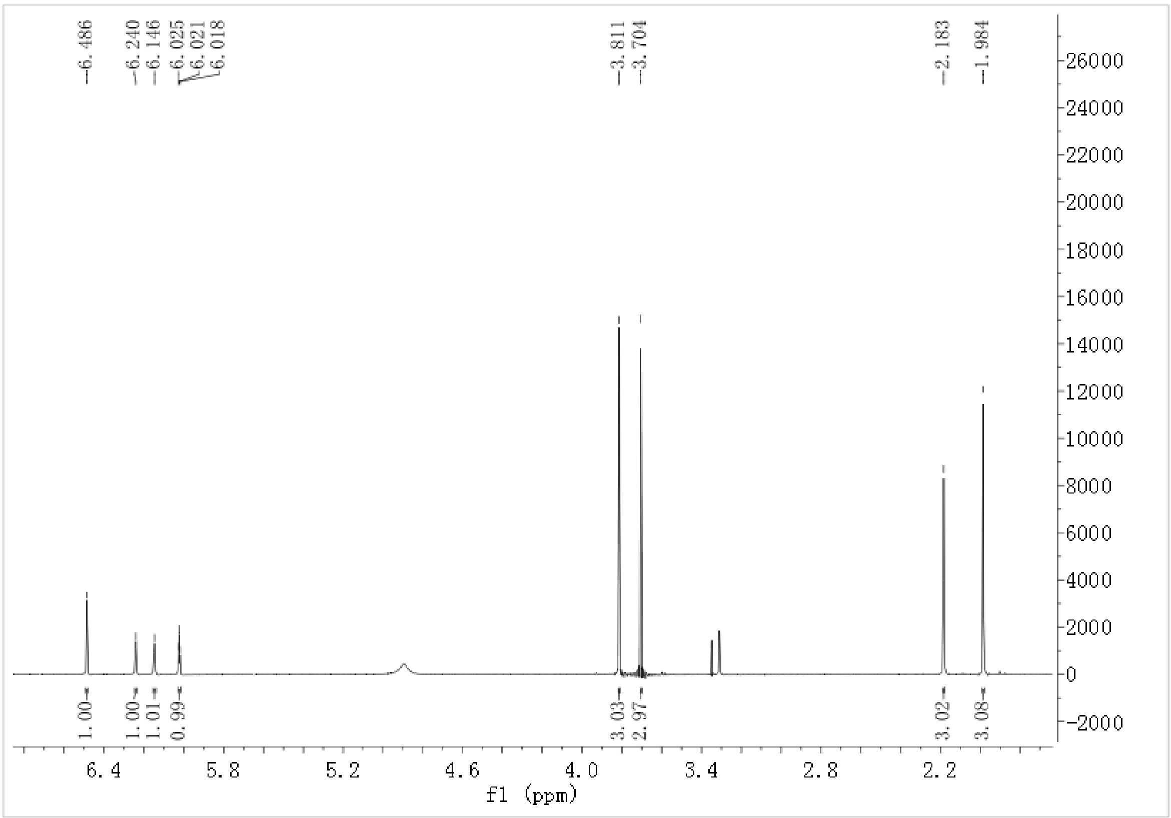

Figure S15. ^{1}H NMR spectrum of compound **3** (CD_3OD).

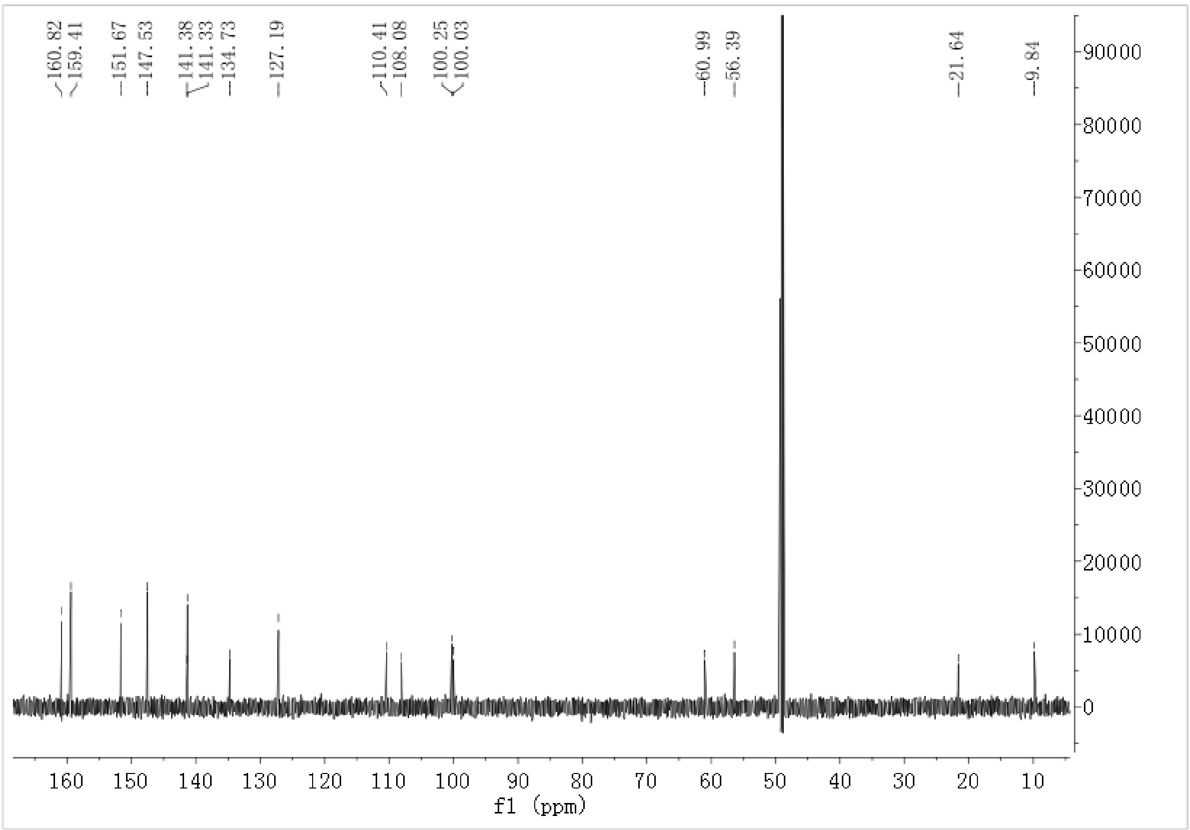

Figure S16. ^{13}C NMR spectrum of compound **3** (CD_3OD).

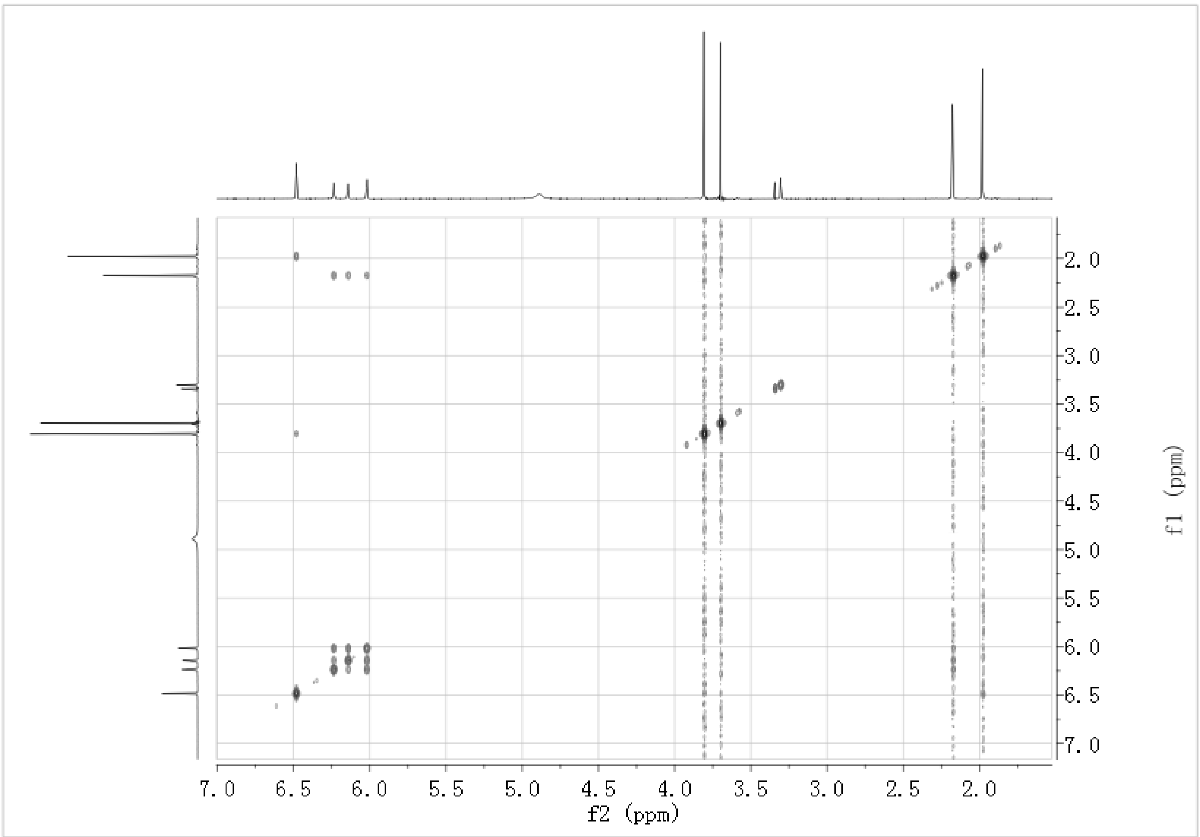

Figure S17. COSY spectrum of compound **3** (CD_3OD).

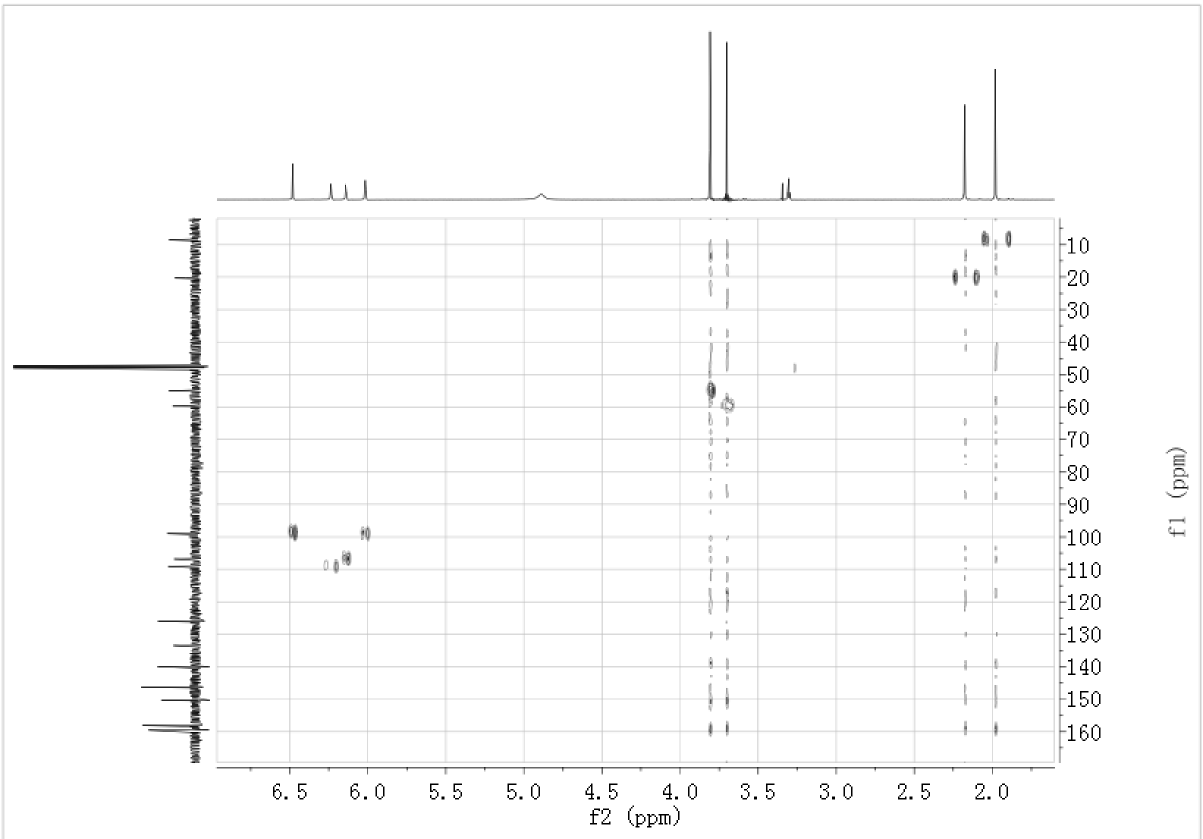

Figure S18. HMQC spectrum of compound **3** (CD_3OD).

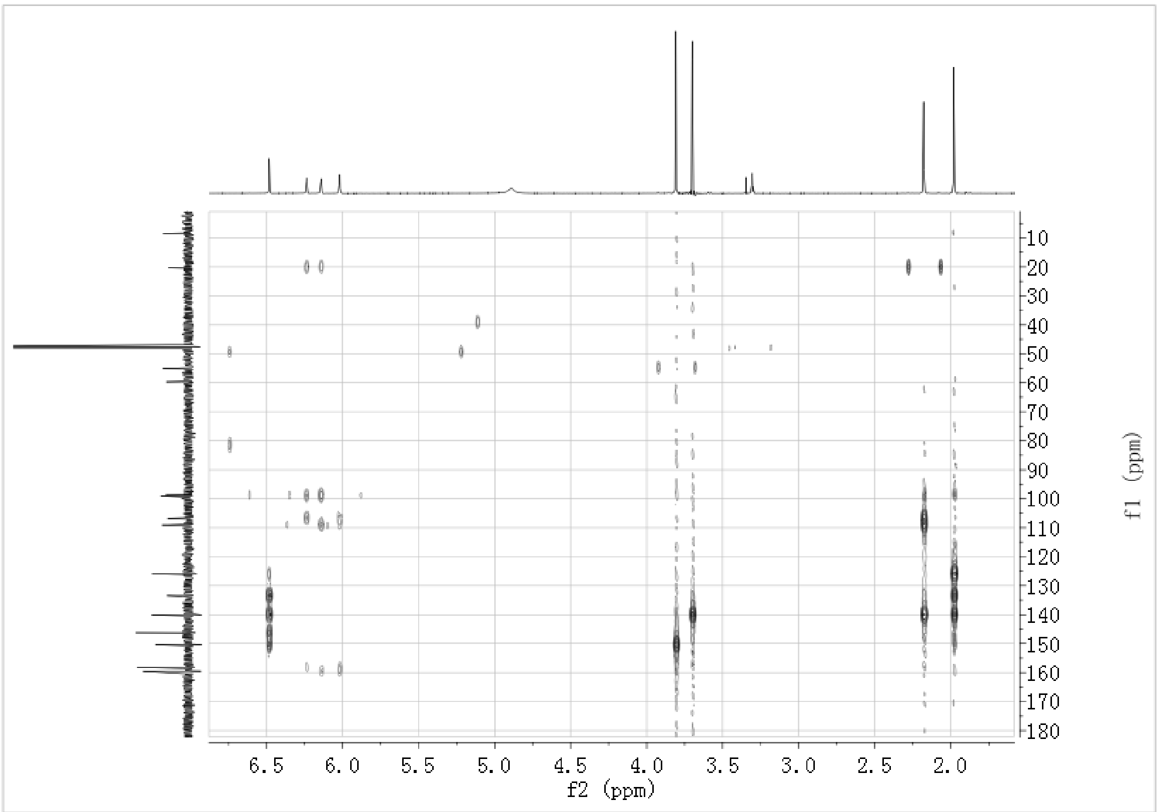

Figure S19. HMBC spectrum of compound **3** (CD_3OD).

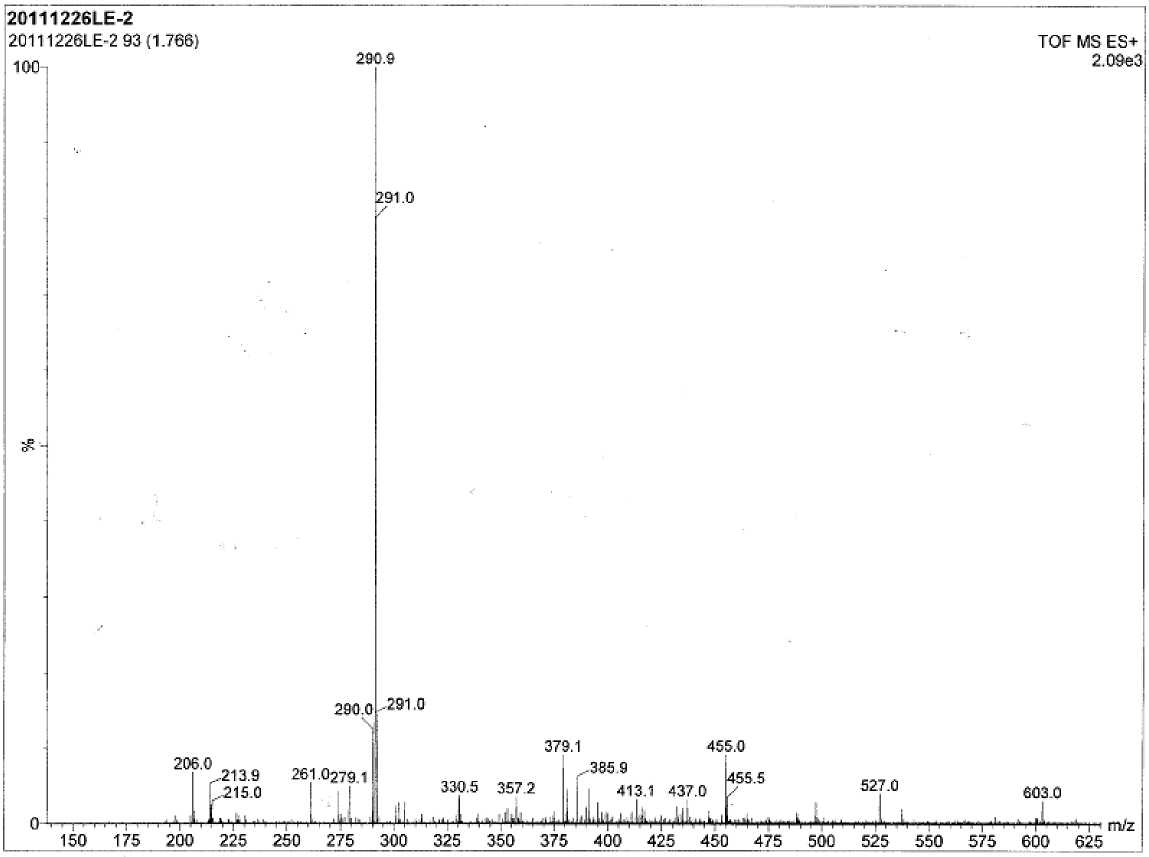

Figure S20. ESIMS spectrum of compound **3**.

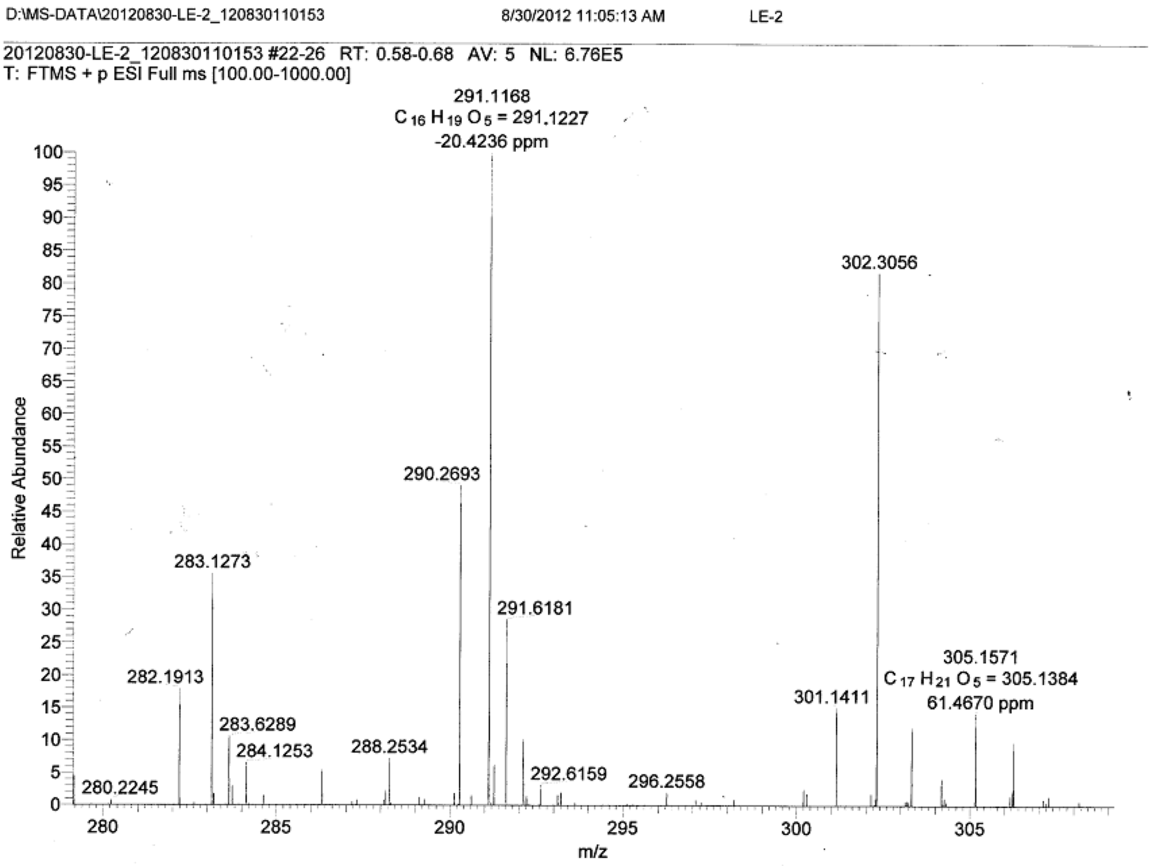

Figure S21. HRESIMS spectrum of compound **3**.
